# Plant phenotyping: from bean weighing to image analysis

**DOI:** 10.1186/s13007-015-0056-8

**Published:** 2015-03-04

**Authors:** Achim Walter, Frank Liebisch, Andreas Hund

**Affiliations:** Institute of Agricultural Sciences, ETH Zürich, Universitätstrasse 2, 8092 Zürich, Switzerland

## Abstract

Plant phenotyping refers to a quantitative description of the plant’s anatomical, ontogenetical, physiological and biochemical properties. Today, rapid developments are taking place in the field of non-destructive, image-analysis -based phenotyping that allow for a characterization of plant traits in high-throughput. During the last decade, ‘the field of image-based phenotyping has broadened its focus from the initial characterization of single-plant traits in controlled conditions towards ‘real-life’ applications of robust field techniques in plant plots and canopies. An important component of successful phenotyping approaches is the holistic characterization of plant performance that can be achieved with several methodologies, ranging from multispectral image analyses via thermographical analyses to growth measurements, also taking root phenotypes into account.

## The conceptual and methodological basis of phenotyping

The terms phenotype and genotype were coined by the Danish plant scientist Wilhelm Johannsen [1,2]. Half a century after Mendel’s experiments on the basis of inheritance and in a time of dispute between the Darwinian and Lamarckian view of evolution, he performed experiments on the heritability of seed size in self-fertilizing beans. Johannsen selected large and small beans of a variety and observed significant difference in seed sizes of the progenies. He concluded that there must be a genetic effect influencing seed size. However, when he selected again within individual plants of the progenies, he could not influence seed size anymore. He concluded that he had selected pure lines for which the phenotype was only driven by environmental effects, such as the seed position on the plant. In his own words Johannsen [2] stated:

“All ‘types’ of organisms, distinguishable by direct inspection or only by finer methods of measuring or description, may be characterized as ‘phenotypes’. Certainly phenotypes are real things; the appearing (not only apparent) ‘types’ or ‘sorts‘of organisms are again and again the objects for scientific research. All typical phenomena in the organic world are eo ipso phenotypical, and the description of the myriads of phenotypes as to forms, structures, sizes, colors and other characters of the living organisms has been the chief aim of natural history, −which was ever a science of essentially morphological-descriptive character….Hence we may adequately define this conception as a ‘phenotype-conception’ in opposition to the ‘genotype-conception’.”

**Figure 1:**
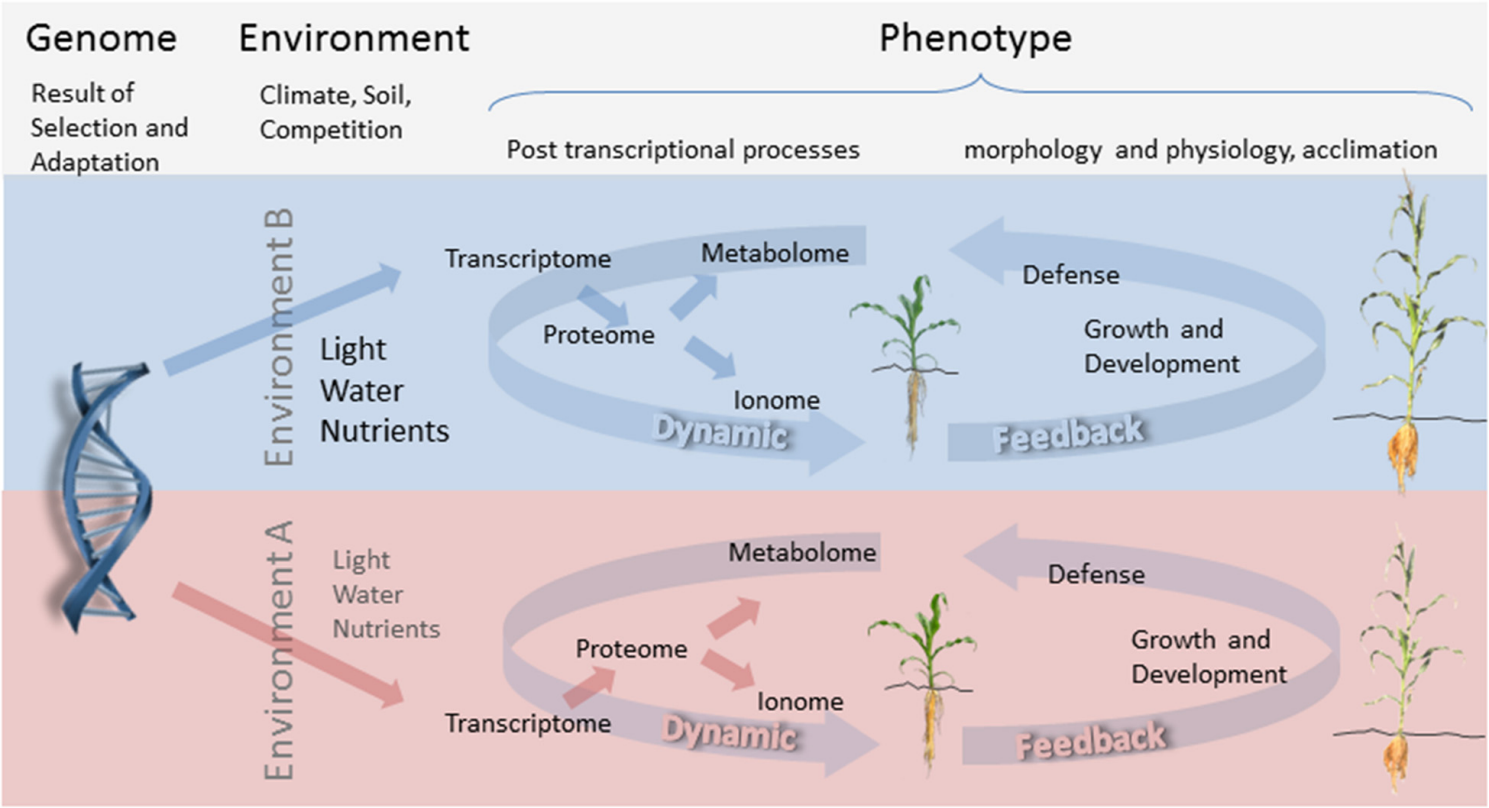
**Relation between genotype and phenotype.** The phenotype is characterized by an enormous amount of processes, functions and structures which are changing during growth and development. Moreover, the regulation of these processes is affected via multiple, dynamic feedback loops by the ever-changing environment. For example: the genotypes available to farmers in form of modern cultivars are the result of selection (by nature and breeders) including biotechnological improvements. While the genotype is comparable to the letters in a book, the interpretation of the genotypic information is affected by the environment. Different genotypes may respond differently to environmental triggers such as limited resources of environment A vs. B. This genotype-by-environment interaction results in different phenotypes which are observable at various organizational levels. A phenotype involves a cascade of processes sequentially altering the composition of the transcribed genes (transcriptome) and their resulting proteins (proteome). These in turn affect the metabolites and ions and act on the development of the plant leading to observable differences in crop physiology and morphology.

Since then, the term phenotype has been used to describe a wide range of traits in plants, microbes, fungi and animals. In plant breeding and quantitative genetics, usually hundreds or even thousands of measurements are performed to select superior individuals or identify regions in the genome controlling a trait. This demands for high-throughput phenotyping, which has been and is still most widely accomplished by quantitative and qualitative assessments (rating) performed by plant breeders. The term ‘phenotyping’ was beginning to be used in the 1960s. In plants, the increasing capabilities of analytical chemistry allowed to broaden the concept of a quantitative analysis of traits to the description of the variability of proteins [3], of metabolic pathways [4] and of other ‘real things’ connected to the character of living plants. From Johannsen‘s description, it is clear that phenotyping – no matter whether in plants, bacteria, fungi or animals – is characterized by an enormous amount of processes, functions, structures, or – most generally spoken – dimensions (Figure 1). In this sense, phenotyping can be considered as far more complex than the analysis of the linear arrangement of genes in the genotype [5]. A comprehensive characterization of the phenotype of any plant – no matter whether it is a model plant or a crop – is far out of reach of the research capabilities of our generation. Therefore, a comprehensive, phenotypic model description of a plant – similar to the model description of an engine – will remain a distant aim of future, ‘Systems Biology’.

Today, high-throughput plant phenotyping refers to the characterization of the whole cascade of changes happening after DNA is transcribed into RNA (transcriptomics) leading to the formation of proteins (proteomics). This cascade from DNA via RNA to proteins, known as the central dogma of molecular biology, determines other plant phenotypic traits, such as metabolites (metabolomics), ions (ionomics), and, last but not least morphological or architectural parameters [6]. According to Guo and Zhu [7], “the purpose of phenotyping is to produce a description of the plant‘s anatomical, ontological, physiological, and biochemical properties”. Throughout the last decade, the terms phenotyping and phenomics have more and more often been linked to non-destructive optical analyses of plant traits based on images [8,9]. Thereby, phenotype analysis is turning its focus back towards the object of interest of Johannsen, but instead of counting, weighing or measuring the length of beans, it is now using image analysis to quantitatively determine Johannsens ‘real things’ [2] of plants in an increasingly holistic and integrative manner. This reorientation began with a study investigating growth of several Arabidopsis genotypes [10]. Utilizing digital imaging to resolve plant rosette area, this pioneering study revealed growth differences between wild-type plants and plants deficient in their photosynthetic capacity within a few days. Today, after 15 years of development within this new scientific field, phenotyping has begun to become a toolbox applicable also to plant breeders to select desirable genotypes for their specific field of interest – be it salt-tolerance in *Triticum* [11], drought-tolerance in barley [12] or maize [13]. Looking back at somewhat more than a decade of non-destructive, image analysis-based plant phenotyping, one can state that the focus of phenotyping has broadened to a certain extent from basic-science oriented analysis of phenotypic differences between a wild-type and a mutant plant from experiments with potted single plants to the analysis of plant plots and canopies in field experiments in the context of plant breeding [14,15] or precision agriculture [16,17]. Of course, a lot of current, image-based phenotyping approaches have originated from the use of non-imaging sensors which have been applied in the field, such as thermography point sensors. Other methods have been introduced from the field of remote sensing, such as the satellite-based calculation of spectral indices.

Based on the pioneering approach of Leister [10], in greenhouses and growth chambers, numerous automated facilities and robots have been set up that allow for the comparison of several hundred plants per day in an automated manner. These setups form the working horse for a lot of scientific investigations in basic research of the public and private sector alike. Moreover, these setups are continuously being refined and their image-processing capabilities form the basis for a next generation of phenotyping platforms that are operating in the field from different carriers such as tractors [18,19], blimps [20,21] or unmanned aerial vehicles [22]. What we are experiencing today is the beginning of a combined use of multiple imaging (or non-imaging, but remote sensing based) technologies for the quantitative description of the performance of plants during their entire ontogeny in their environment (Figure 1). In this early phase of computer-vision-based plant phenotyping methods, concepts and approaches are proposed, which allow for a characterization of the overall performance of a plant in its given environment. Of course, these developments would not have been possible without decades of pioneering work in photogrammetry and remote sensing, which is the science and technology of obtaining information about physical objects and the environment through the process of recording, measuring and interpreting imagery derived from non – contact sensor systems [23].

**Figure 2:**
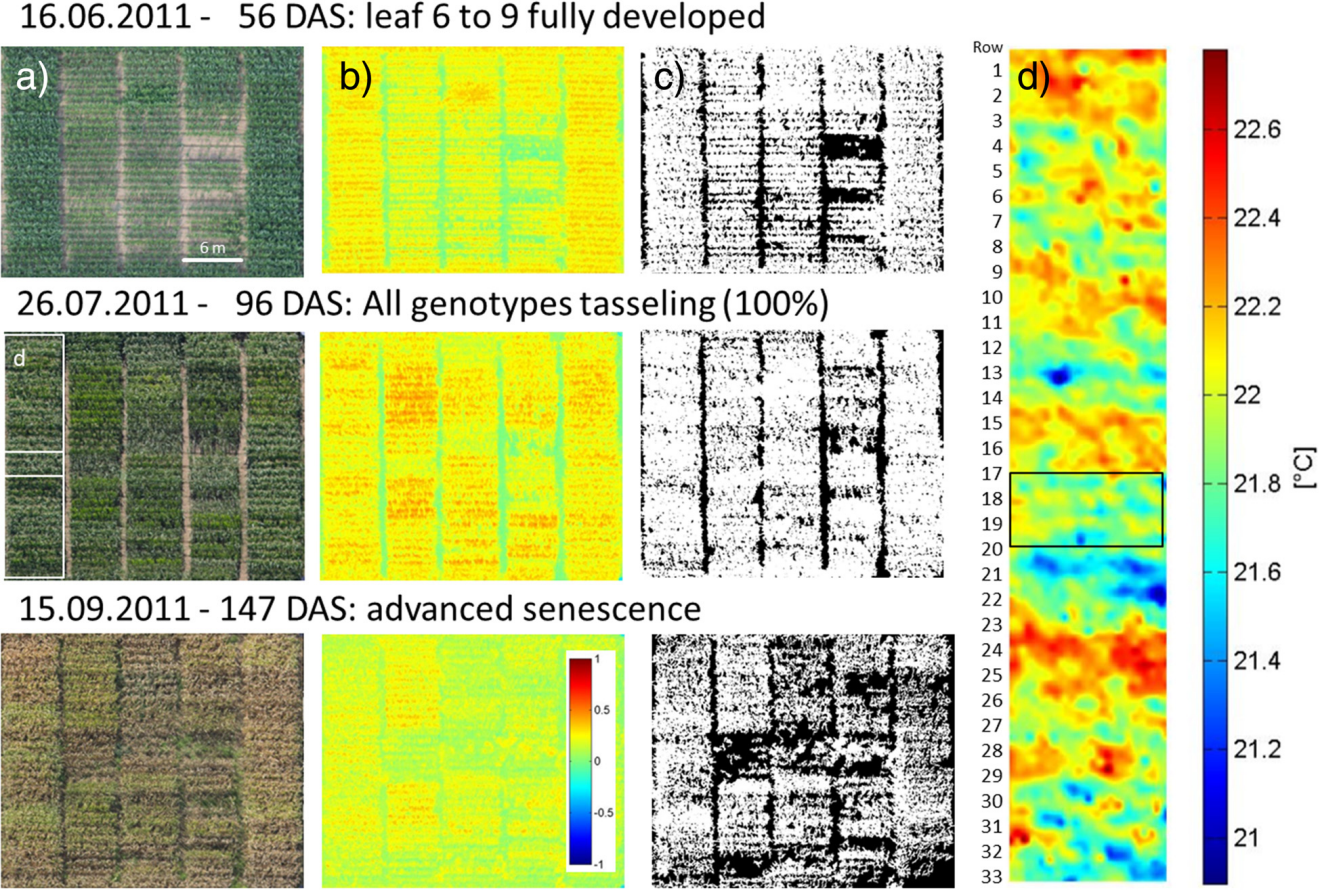
**Images related to core methods of image-based plant phenotyping in the field at three characteristic ontogenetic stages typically investigated for breeding purposes in maize.** Images are taken from a maize field experiment in Germany [24] at several ontogenetic stages from an altitude of 300 m. **a)** RGB image, **b)** NDVI-image, **c)** canopy cover segmented from NDVI-image, **d)** thermography image of a subsection of the area shown in the image from 26.07.2011. The graph shows a set of maize genotypes at an early growth stage when canopy cover is different (16.06.2011), at a growth stage when the canopy of all genotypes is closed but leaf greenness and tassel appearance differs between genotypes (26.07.2011) and at a late, senescent stage when different levels of senescence or stay green can be observed (15.09.2011).

**Figure 3:**
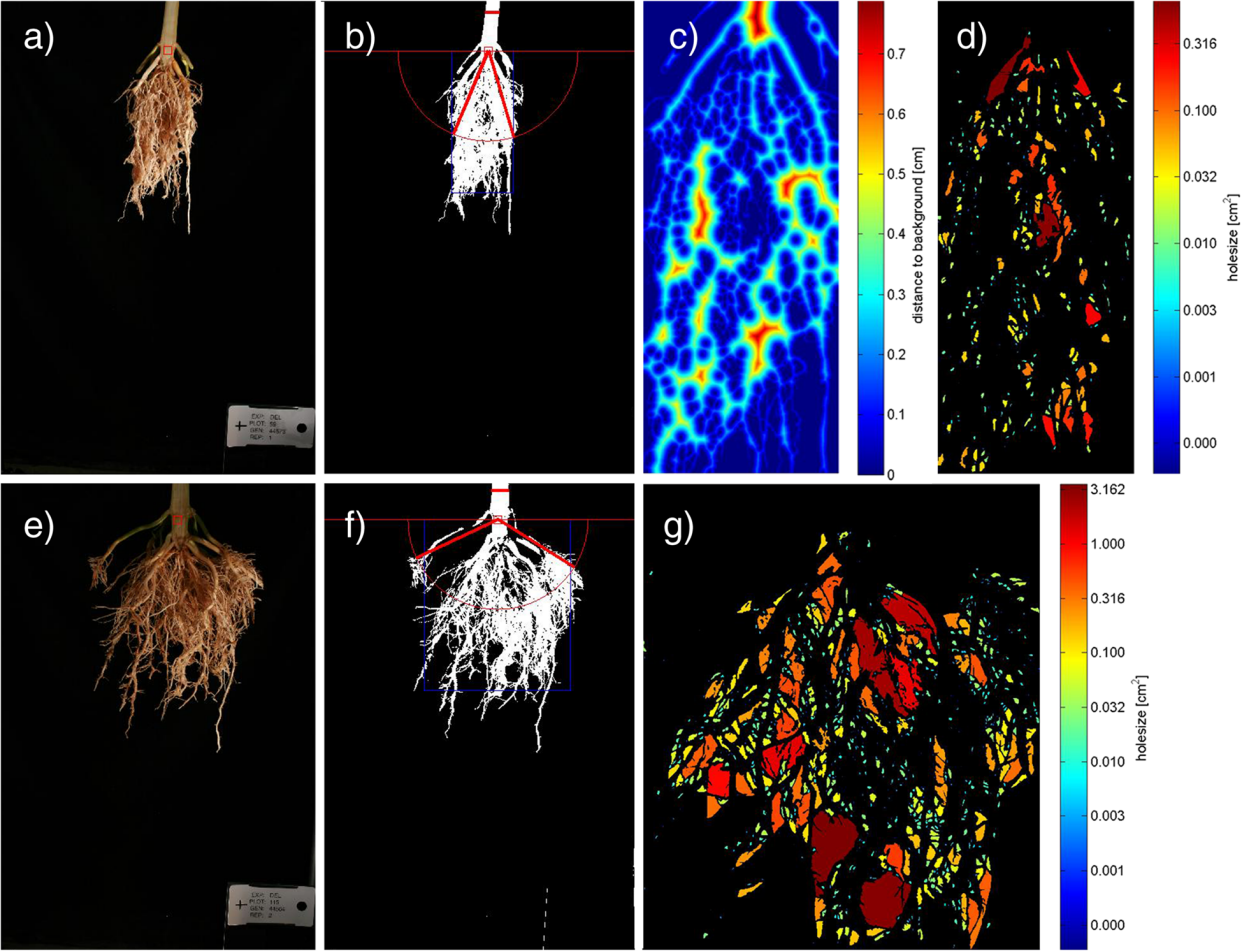
**Example images related to shovelomics, a method for field phenotyping of crop root systems:** Two field grown maize genotypes (top, bottom) with contrasting root angles, identified with the software REST (Root Estimator for Shovelomics Traits) [119]. Original image **(a, e)**, resulting area of interest containing about 90% of the root system (**g**, **f**; blue box) and the opening angle of the root system (**b**, **f**; red lines); visualized thickness of root clusters **(c)**; and whole sizes **(d, g)**, related to root branching.

To get a more comprehensive overview on the achievements of plant phenotyping, it may be helpful to structure the state of the art into four main classes of methods that are currently being used (Figures 2 and 3). These methods are related a) to the spectral reflectance and absorbance of leaf, plant and canopies, b) to the plant or canopy temperature and derived indicators for transpiration and water status, c) to size, morphology, architecture or growth of plants or their canopies and finally d) to the architecture of the root system analyzed in the lab and in the field. Distinguishing these four methodological classes only partly reflects structural or functional core categories of the plant, but it shows the current activity of the plant phenotyping community driven by available sensor technologies and analysis methods. In all technologies and for all research aspects, it is crucial to attempt a precise positioning of the required sensors and to perform reliable measurements at high-throughput and high precision to advance our capabilities and to arrive at a more holistic characterization of plant or crop performance [14]. Future phenotyping approaches will most probably analyze several aspects of plant performance at the same time, potentially using multiple sensors, thereby resolving complex traits, such as demonstrated e.g. by Liebisch et al. [24].

## Spectral assessment of plant shoots and canopies

Spectral indicators used for plant trait detection and phenotyping range from simple ratios calculated from responses at two wavelengths [13,16], via normalized indices [13,16] (example discussed below) to very complex equations and algorithms [25,26]. A very immediate indicator of plant performance is its leaf color. Our eye is highly sensitive to different shades of green. Leaf greenness is determined by genotype specific properties such as content and development of leaf chlorophyll, by plant health and by leaf morphological characteristics such as thickness and surface structure. Leaf greenness changes according to plant development, is affected by plant nutrition and environmental stresses such as cold, heat and drought stress. The most frequently used indicator for leaf greenness in remote sensing is the normalized difference vegetation index (NDVI) [27,28], which exploits the difference between reflectance in certain regions of the visible light spectrum (VIS), where absorption of chlorophyll is maximal, and in the near-infrared part of the spectrum (NIR), which is not affected by photosynthesis. Most often, reflectance and absorption in the visual range are narrowed down either to the red or to the blue region of the spectrum, where chlorophyll and light harvesting antenna pigments are absorbing maximally.

The exact calculation of NDVI with respect to the wavelengths used depends on the objectives of the study and on the sensor, but in general it is calculated as NDVI = (NIR-VIS)/(NIR + VIS), thereby normalizing the difference between reflection in the visible (VIS) and the near-infrared range (NIR) to the sum of reflected light in both ranges. For the visible range often red or blue bands are used for detection of NDVI [24,27,28]. This allows for a comparison of plants or canopies in different illumination situations, such as in a field during a somewhat cloudy day or in order to compare between measurements taken at different days (Figure 2). With respect to plant phenotyping, NDVI has been applied to study phenology changes in crops [29-31], to study vegetation ecology [32,33], stress [34,35] and nitrogen status [36-38]. Remote measurement of leaf greenness on the canopy scale is affected by the angle of the optics towards the canopy, by illumination conditions and by canopy characteristics such as canopy height and leaf angle distribution. Recent studies in wheat and maize correlate NDVI with important crop properties such as biomass [39,40], chlorophyll content [41,42] and nitrogen status [34,43]. The development of NDVI during a season or an extended period of time can be used to investigate traits important for breeding such as stay green [44,45] and growth rates [46].

It has to be pointed out that NDVI is just one of a huge number of spectral indices that can be utilized for remote characterization of plant performance [16,26,47,48] in the field and in laboratory studies. Other indices, such as the ‘modified chlorophyll absorption ratio index’ take spectral components of green light into account and provide thereby some information about the density of the canopy, since green light is also reflected to the sensor from deeper layers of the canopy, penetrating the top layers. For some phenotyping applications, the analysis of canopy coloration is performed not from purely reflected sunlight, but the plant canopy is actively illuminated. In agricultural management, sensors such as the ‘GreenSeeker^TM^’ (NTech Industries, Inc., USA) or ‘Crop Circle^TM^’(Holland Scientific Inc., USA) have been introduced to the market years ago: There, the canopy is actively illuminated by hand-held or tractor-mounted devices that also perceive and interpret the reflected radiation. Based on the calculated NDVI or greenness indicator, fertilization of the investigated crop patch is performed (low greenness – more nitrogen fertilizer required), taking species-specific crop models into account.

The analysis of a certain part of the visible light spectrum following an induction by active illumination is also the foundation for phenotyping analyses based on chlorophyll fluorescence of a canopy [49]. Chlorophyll fluorescence has been used to describe the performance of the photosynthetic apparatus from the analysis of light emitted at longer wavelengths and at later times (between μs and a few minutes) following up on an illumination pulse. It has been noted in the 1930s [50] that due to electron transfer processes within the photosystem, characteristic intensities of photons are emitted that can be used to derive potential photosynthetic yield, quantum efficiency of photosystem II (φ_PSII_) and other parameters. Chlorophyll fluorescence has successfully been used in phenotyping studies in the laboratory [51-53] and in the field [54]. Traditionally, chlorophyll fluorescence has been measured using hand-held devices. It was successfully applied to select maize with greater cold tolerance of the photosynthetic apparatus [55]. In the field, laser-induced chlorophyll florescence was for example used to determine biomass and nitrogen status in oilseed rape [56]. A problem of actively remotely sensed chlorophyll fluorescence is that it needs a true saturating light pulse in order to determine crucial parameters, such as φ_PSII_, which can be achieved by applying a laser from a long distance. Alternatively, very high spectral resolution in ideally sub-nm range is used today for passive estimation of chlorophyll fluorescence from solar reflectance spectra by for example the Fraunhofer line depth technique (FLD) [57-59].

## Thermography-based investigations of transpiration in the soil-plant-atmosphere continuum

Another important line of research utilizes sensors that detect canopy temperature from long wavelength infrared radiation according to the relation between body temperature and the light spectrum emitted from that body [60,61]. Since plant tissues are cooled by transpiration of water, canopy temperature can be linked to transpiration rates, if the temperature of the canopy and of the surrounding environment can be analyzed precisely enough. Therefore, thermal imaging offers a large potential for non-destructive measurement of plant water status for irrigation management and for phenotyping [62] in the context of stress tolerance or drought stress avoidance [63]. Yet, it is far from being trivial to interpret plant temperatures correctly, since they depend strongly on the microclimate of the plant stand and they need to be balanced carefully with reference temperatures of non-transpiring and/or fully transpiring canopies in close spatial and temporal vicinity (see [61] for more details). Another constraint of thermal imaging is the high temporal and spatial variability caused by a) environmental conditions changing rapidly in the field e.g. on cloudy days [63-65], and b) different canopy densities of different genotypes that can lead to non-comparable microclimatic conditions in multi-plot field experiments [62]. Plant canopy temperatures may also be strongly affected by differences in development of examined genotypes. Early flowering and a concomitantly earlier start of senescence e.g. affect canopy temperature by reducing transpiration per square meter from an ageing canopy, which carefully needs to be taken into account. Plant density differences might be caused by different germination rate resulting from field variation in soil properties, by genotypic differences or by different sowing density. The effect of background temperatures can be separated by normalization to background temperatures [63], but other climate parameters such as radiation or wind speed affect leaf temperature as well [66] and their quantitative effect is not well understood under field conditions. Nevertheless, it has to be pointed out that thermography is a powerful, integrative tool to differentiate between phenotypes, especially if it is used to test the overall effect of precisely defined physiological aberrances on certain plant genotypes in the field. An example for such an application is the detection of early stress symptoms of plant diseases [67] which affect transpiration. Thereby, thermography facilitates phenotyping for disease-resistant plant genotypes – one of the most important plant breeding aims in all major crops.

## Optical analysis of aboveground plant size, organ and canopy growth

The most direct, overall plant performance indicator – at least during the ontogenetic phase of vegetative development – is the growth of plant biomass or plant size. As mentioned above, this is often monitored in a global way by assessing the number of pixels, which an individual plant or the total canopy of an experimental plot is covering within an image of calibrated size [10]. Such methods have been successfully applied in the laboratory to assess the performance of Arabidopsis [68], tobacco [69] or cereal grain crops [11,12]. All global players of the agro-biotech business do have such monitoring platforms, with which they test differences between genotypes [51] or effects of plant protection or plant strengthening substances applied to a crop of interest. Size analysis of the plant is not as straightforward as it may seem since the precision of the measurement depends strongly on the orientation between canopy and sensor, on the precise distinction between object and background and other pitfalls of image analysis in the context of plant phenotyping [70].

In field experiments, plant size is not only estimated from top-view images, but – especially for monocotyledonous crops – by analysis of canopy height from measurements of light barriers mounted on tractors that analyze the top level of a canopy as the tractor pulls the light barriers along the seeded rows of the crop [19,71]. Other field applications comprise the analysis of canopy cover (CC), which simply refers to the fraction of the ground that is covered by the canopy [21,24,72-74]. The CC trait can be used to detect temporal and genotypic differences and it is linked to important plant traits such as early vigor and senescence that have long been used for crop breeding, turning CC into one of the key traits for ‘next generation phenotyping’ [15]. CC can be calculated from digital images with a red, green and blue channel (RGB) or NDVI images, segmenting the green plant from the non-green background or even from images that assess chlorophyll fluorescence. In principle, it is also possible to determine plant shape, number of leaves, and structure of the canopy or leaf area index from such images – especially when they are used to reconstruct the 3D-shape of the canopy either from multiple images or from scanning the canopy. 3D-reconstruction already works in the lab [75-77], but is challenging to be reliably performed in the field [78]. Yet, with the increase of computing power and with modern imaging capacities of unmanned aerial vehicles [16,22] and other devices that are capable of generating plant images in the field from multiple perspectives, it should be possible to advance enormously in this area in the near future. Then, automatic counting of tiller numbers, ear densities, fractions of damaged leaves and other traits relevant in classical breeding programs can be performed. Also, the dynamic development (when does a plant grow how intensely) and the relation of plant growth and environmental parameters (which genotypes grow best at certain temperatures) will form an important focus of next generation phenotyping. In a proof-of-concept study Grieder et al. [79] investigated wheat genetic variation in growth response to temperature using image based phenotyping in the field.

## Root phenotyping at high**-**throughput in controlled conditions

Root phenotyping is as important as shoot phenotyping, since the performance of any plant strongly depends on its root architecture and function [80-82]. The added value of root phenotyping becomes obvious e.g. in breeding programs, in which it is shown that root traits sometimes have a higher heritability than the aboveground target trait (e.g. grain yield). Good examples of the high importance of root architecture are a) the benefit of shallow rooting in phosphorous-poor soils, which maximizes P uptake from the topsoil [83] and b) the benefit of aluminium tolerance in acidic tropical soils which enhances deep rooting [84].

For methodological reasons, root phenotyping capabilities have been developed in the laboratory first and are now evolving towards field applicability – in a similar manner, but with some temporal delay compared to shoot phenotyping capabilities. Laboratory-based methods to study root growth were recently reviewed by Zhu et al. [82]. Root phenotyping platforms and methodologies usually combine some degree of automation with imaging and image processing. To facilitate the inspection of roots, special care has to be taken how to cultivate plants in a way that allows for normal plant development and for access to the root. The most basic and hence most widely used systems to observe roots are based on soil-free growth media. There, the root either grows in paper rolls [85] on the surface of germination papers [86,87], or gels [88-90], in air regularly sprayed with nutrient solution [91] or in aerated aqueous solutions [92]. Another version of hydroponics, which includes some degree of mechanical resistance, is to cultivate roots in transparent plexiglas nail board sandwiches filled with 1.5 mm glass beads through which a nutrient solution is circulated [93]. In all of these systems, total root length, branching angles and other parameters are determined, using manual measurement, visual rating or imaging. Imaging needs to be performed with high resolution scanners or cameras to be able to resolve lateral roots for image processing. The basic global evaluation of images extracts root length. Often, individual root diameters are used as decision criterion to distinguish between the main roots and their lateral branches [86,94] typically upon usage of the software WinRhizo [95,96]. With the development of suitable software such as SmartRoot [97] that allows for topology analyses, root system architecture can be analyzed in detail for branching angles etc. and growth kinematics of individual roots within the root system [87]. Still substantial manual input is required for such analyses [87]. Thus, there is still the need for significant improvement of image processing, even for soil-free systems in which roots are comparably easy to detect. In case of soil as growth medium, image processing becomes even more challenging. However, more natural systems like soil-filled rhizotrons or growth columns are indispensable to study the interaction of roots with edaphic factors. For example, soil compaction or the effects of drying soil are difficult to establish in soil-free systems.

Rhizotrons or columns, filled with soil or other growth substrates, enable a direct inspection of roots along a transparent wall [98] or within a small soil column by using x-ray based computed tomography [99,100] to visualize the 3D-configuration of roots. The most advanced versions of these systems combine large soil volumes with high**-**throughput and automation. These are the soil-filled 2D rhizotrons of the GROWSCREEN-Rhizo platform with a rooting depth of 90 cm [101] and 25 cm diameter-by-100 cm growth columns in combination with μCT imaging at the Hounsfield facility of University of Nottingham [102]. Lysimeters in form of tall columns that are placed with a distance to each other in order to simulate a planting density as under field conditions are well suited to get an indirect measure of rooting depth and water uptake by means of regular weighing [103]. Such systems can serve as an excellent bridge between controlled conditions and real field conditions. Other methodologies with the potential to study root system architecture and functioning in the future are nuclear magnetic resonance [104-106], neutron radiography [107] and positron emission tomography [108], which allow segmenting the root from the surrounding substrate.

## Root phenotyping in the field

Due to the hidden nature of roots, it is extremely difficult to assess them optically in the field – unless one is digging them out or one approaches them using a tunnel. Therefore, the most widely used traditional methodology to study roots in the field is the so-called ‘trench profile’ method, in which soil is carefully removed from the side, often using fine brushes, and in which the root system is then sequentially revealed and drawn layer by layer from successive profile walls [109-111]. In other approaches, soil cores are taken in order to sample vertical root length densities or weights, sometimes using semiautomatic extraction methods [112,113]. A far more rapid method to evaluate the maximum rooting depth from soil samples is the core break method, developed by Bohm [114]. In this method, soil cores of up to 2 m length are broken into sections of 10 cm to determine the maximum rooting depth, corresponding to the depth of the last interface at which a root is observed [85].

Another promising ‘field-technique’ widely practiced is the analysis of excavated upper parts of the main root system [115-117]. This method is termed “Shovelomics” [117] and is performed by excavating a few liters of soil with one crop plant in the center of the surface. Soil is gently washed away from the top part of the root system and the core skeleton of the main root branches is then analyzed for parameters such as root angles and densities (Figure 3). Analysis methods comprise a wide range of different techniques from simple rating and counting [117] to imaging in combination with custom image analysis software [115,118,119], allowing to measure basic root characteristics related to branching, root dimensions and structure (Figure 3). The shovelomics method still has to prove its value for trait-based selection by delivering new yield-related traits that cannot be measured sufficiently above-ground.

Other techniques applicable to field studies are based on so-called mini-rhizotron systems which consist of plexiglas tubes inserted into the soil, in which a small camera or a scanner, is inspecting the surrounding root-soil continuum (see review by Johnson et al. [120]). Limited numbers of genotypes may be monitored using these mini-rhizotrons [121,122]. Several other, indirect methods were proposed and used to analyze root system architecture or overall root performance, such as root pulling resistance [123] or the analysis of leaf abscisic acid content [124]. Total root mass has been proposed as a trait to be measured by electrical capacitance measurements that analyze the response behavior of currents applied to one electrode inserted at the base of the stem and to another electrode in the rooting substrate [125,126]. This method has been used in high**-**throughput analyses of root mass in the field [127,128], but recent studies indicate that the “root capacitance” may be more related to the cross sectional area (or circumference) of the root at the soil [129] or solution surface [130]. These observations cast some doubt on the reliability of this otherwise promising approach to explore root-soil interactions and root phenotypes based on electrical properties. Clearly, the intensity of water uptake is related to transpiration (and thereby can be assessed with thermography as shown above) and it alters electrical properties of the soil in a way that can be determined by changes of the total electrical resistivity of soil situated between two electrodes [131]. Maybe, a dynamic analysis of ion and water content in the rhizosphere, which can be performed on the basis of electrical analyses, will become an element of our capabilities to characterize an important trait of the multidimensional plant phenotype: water and nutrient uptake. Of course, this does not depend only on the root system architecture, but also on intrinsic hydraulic properties and the uptake and transport efficiency of tissues [132,133]. Therefore, the set of methods to analyze overall indicators of plant performance in plant phenotyping will increase surely in the near future, allowing then to obtain a more and more holistic view of plant performance.

## Conclusion

The field of plant phenotyping is still under rapid development at the moment. Image-based plant phenotyping is beginning to prove its value not only in basic science, but also in crop breeding and precision agriculture, providing a quantitative basis of the description of plant-environment-interactions. Key to the success is the ease and applicability of modern image analysis approaches that are applied at multiple points in time throughout crop development, thereby allowing for cost-efficient high-throughput phenotyping at appropriate ontogenetical stages. Since the potential of image analysis in the context of plant phenotyping is far from being adequately exploited, the scientific field of plant phenotyping can be expected to continue prospering throughout the coming years.
